# A strong construction of S-box using Mandelbrot set an image encryption scheme

**DOI:** 10.7717/peerj-cs.892

**Published:** 2022-09-09

**Authors:** Mazzamal Aslam, Saira Beg, Adeel Anjum, Zakria Qadir, Shawal Khan, Saif Ur Rehman Malik, MA Parvez Mahmud

**Affiliations:** 1 Computer Science, COMSATS Institute of Information Technology, Islamabad, Pakistan; 2 Department of Information Technology, Quaid e Azam University, Islamabad, Pakistan; 3 School of Computing Engineering, University of Western Sydney, Penrith, Australia; 4 Information Security Institute, Cybernetica AS, Tallinn, Estonia; 5 School of Engineering, Deakin University, School of Engineering, Australia

**Keywords:** Substitution-box, Chen chaotic system, Mandelbrot set, Cryptosystem, Image encryption

## Abstract

The substitution box (S-box) plays a vital role in creating confusion during the encryption process of digital data. The quality of encryption schemes depends upon the S-box. There have been several attempts to enhance the quality of the S-box by using fractal chaotic mechanisms. However, there is still weakness in the robustness against cryptanalysis of fractal-based S-boxes. Due to their chaotic behavior, fractals are frequently employed to achieve randomness by confusion and diffusion process. A complex number-based S-box and a chaotic map diffusion are proposed to achieve high nonlinearity and low correlation. This study proposed a Mandelbrot set S-box construction based on the complex number and Chen chaotic map for resisting cryptanalytic attacks by creating diffusion in our proposed algorithm. The cryptosystem was built on the idea of substitution permutation networks (SPN). The complex nature of the proposed S-box makes it more random than other chaotic maps. The robustness of the proposed system was analyzed by different analysis properties of the S-box, such as nonlinearity, strict avalanche criterion, Bit independent criterion, and differential and linear probability. Moreover, to check the strength of the proposed S-box against differential and brute force attacks, we performed image encryption with the proposed S-box. The security analysis was performed, including statistical attack analysis and NIST analysis. The analysis results show that the proposed system achieves high-security standards than existing schemes.

## Introduction

Nowadays, data security is one of the challenging tasks across the globe, where everyone is linked to others through some sort of communication medium. The data such as audio, video, images, and documents transmitted through different mediums need to be secure from eavesdropping and malicious nodes. Data confidentiality is the main objective of data security. Various cryptographic approaches are employed to ensure data security. In 1949 C.E., Shannon (1949) suggested the development of cryptographically strengthened systems with two fundamental properties of confusion and diffusion. Block ciphers are essential in the construction of cryptosystems in modern cryptography. They place a high value on the strength of replacement boxes (S-boxes) (Razaq et al., 2021; Razaq et al., 2021). The S-boxes are used to aid with data distortion and to strengthen encryption (Khan & Asghar, 2018; Ullah, Jamal & Shah, 2018). S-box is essentially a series of permutations that map m-bits input to n-bits output. A single non-linear conversion function in the S-box performs the uncertainty bit; as a result, a large non-linear S-box is needed. The need for a good S-box in cryptographic techniques is a topic of considerable concern in the research community. The S-box has taken a significant amount of time and work to build as discussed and observed from the literature (Aboytes-González et al., 2018; Bibi et al., 2018; Khan, Masood & Alghafis, 2020; Razaq et al., 2021; Khan, Ahmed & Saleem, 2019; Özkaynak, Çelik & Özer, 2017). Vectorial bent functions are the highest possible high nonlinearity score for even n-bits dependent S-boxes and can only occur for *m* = *n*/2 (Zhang et al., 2018). The production distributions of all vectorial bent function derivatives are identical, but they do not match to a balanced S-box (Juremi et al., 2019). The performance of block cipher encryption schemes depends entirely on the S-box architecture process. Chaotic maps, power polynomials, DNA sequences, TDERC sequences, Galois field, machine learning, inversion mapping, Gaussian noise, and pseudorandom number generator approaches were the most utilized S-box construction methods (Razaq et al., 2021; Khan, Masood & Alghafis, 2020; Khan, Ahmed & Saleem, 2019). AES, APA, Gray, Skipjack, Xyi, and Residue Prime (RP) S-boxes are present in the literature (Khan, Masood & Alghafis, 2020). The approaches described in the literature are complex algebraically, but they also have superior cryptographic properties. The properties of the S-box are commonly used to determine the strength of an encryption form.

### Contributions

• The Mandelbrot set is used in this article because it contains an intricate structure that arises from a basic description. A slight change in the parameter can modify the shape of the Mandelbrot set.
• The initial condition sensitivity shows its chaotic behavior. The slight change in the initial parameters of the Mandelbrot set completely changed the output value.
• In literature, chaos-based cryptographic protocols could be resilient to cryptanalytic attacks rather than mathematical encryption protocols. Therefore, we used Chen’s chaotic system to create diffusion in the offered cryptosystem.
• The proposed scheme also deceives all possible linear cryptanalysis attacks such as a chosen-plaintext attack, chosen-cipher attack, and known-plaintext attack.
• Security proof of our scheme will be presented in preferable security assumptions, such as non-linearity, strict avalanche criterion, differential probability histogram analysis, correlation measures, entropy analysis.

Using fractal-based sequence property, we generate an S-box with maximum non-linearity by changing the parameters. The typical Mandelbrot set is defined as:

(1) \begin{eqnarray*}f:{z\leftarrow z}^{2}+c.\end{eqnarray*}

Researchers have also produced a variety of methods for S-box analysis (Khan et al., 2018; Picek et al., 2014), *i.e.,* non-linearity methods, strict avalanche criteria, probability methods (linear approximation (LP), differential approximation (DP)), and bit independence criteria (BIC) are among these methods and criteria (Razaq et al., 2021; Khan, Masood & Alghafis, 2020; Khan et al., 2018; Picek et al., 2014). Recent studies have shown that chaos-based cryptographic protocols could resist cryptanalytic attacks rather than mathematical encryption protocols. Therefore, we have utilized Chen’s chaotic system to create diffusion in the offered cryptosystem. Moreover, the cryptographic strength of the proposed cryptosystem is dignified by some formal analysis, such as randomness analysis, National Institute of Standards and Technology (NIST) test, differential attack analysis, and pixel similarity-based analysis. The rest of the article is classified as: ‘Literature Review’ presents the literature review; in ‘Proposed Cryptosystem’, we provide a specific technique for key generation and the encryption scheme of the proposed image cryptosystem. In ‘Formal Modeling of Mandelbrot Set S-box’ and ‘Performance Analysis and Results’, a detailed security analysis and comparison of the proposed cryptosystem are mentioned. Finally, in ‘Statistical Analysis’, this study’s conclusion and future work are provided.

## Literature review

Researchers in the world of information technology has put in much effort over the last decade. Image encryption has piqued the interest of information security specialists. Since the images contained a large amount of data, it is challenging to encrypt the information utilizing fundamental mathematical processes properly. Researchers have utilized several non-linear functions to effectively encrypt digital images, with chaotic behavior being the most used non-linear method. Shannon (1949) suggested a relatively strong mechanism, which is a mixture of diffusion and confusion, in addition to chaotic processes. Diffusion may be achieved in various methods, and the S-box is one of the non-linear components that can be used to add diffusion to a simple image. Several techniques for encrypting single and multiple S-boxes have been proposed.

### S-boxes

The image encryption method uses two types of s-boxes: static and dynamics. Static s-boxes are less secure than dynamic s-boxes. Dynamics s-boxes are more efficient and usable than static s-boxes because of the prior additional keyspace.  Lu, Zhu & Deng (2020) proposed a single dynamics scheme for the construction of the S-box. They use two turns of chain substitution and one turn of pixel permutation. An image encryption scheme was proposed, which depends upon block permutation for S-box construction, in Ping et al. (2018). The chaotic operation was used for the confusion and diffusion purpose. Genetic algorithms approaches were used to solve the challenge of S-box creation. In order to meet the non-linear behavior and other strength criteria, Farah, Rhouma & Belghith (2017) employed a genetic algorithm optimization to enhance the designed S-box continuously. On the other hand, soft computing techniques are not fast enough to construct dynamic S-boxes in real-time. In Javeed, Shah et al. (2020), a novel method of s-box construction was proposed. The system of equation Rabinovich Fabrikant (RF) is chaotic and more dynamic due to its non-linear feature. This system is ideally designed to design a non-linear block cipher component.

### Mandelbrot set

 Agarwal (2020) presented the CFF technique, composite fractal function (CFF) is the combination of two distant Mandelbrot sets having a single threshold value. A z-scanned fractal pattern is used for increasing randomness in the image by applying random fractal matrix and Henon map-based plain image pixel scrambling. The analysis showed that the proposed CFF method exhibits all required chaotic features. In Sani, Behnia & Akhshani (2021), the S-box was generated through a complex map. They use Julia set for creating confusion and diffusion in their s box and cryptosystem. The real part of the Julia set was used for the generation of S-boxes, and the imaginary part was used for diffusion in their algorithm. Experimental results show that their algorithm satisfies most of the required criteria, but there is still some limitation to secure image encryption algorithm. Their result shows there is less robustness in the proposed S-box algorithm. In Hasanzadeh & Yaghoobi (2020), another fractal-based scheme was proposed in which the Julia set and three-dimensional chaotic map for image encryption were used. At first, they encrypted images with Julia set by shuffling image layers, and then the encryption is done with the Julia set by doing the same shuffling process. The confusion and diffusion property of the algorithm enhanced the robustness of the proposed system. Another fractal-based scheme was proposed by Zhang, Hao & Wang (2020). They generated a keystream from the Julia set created confusion in image layers and then used the Mandelbrot set to create another keystream. After generating two key streams, they shuffled them and created confusion in the image by the updated keystream. The constructed Julia set and Mandelbrot set created confusion until complete disorder in the original image, followed by diffusion using the XOR operation. A key-dependent permutation over finite elliptic curve scheme proposed (Ibrahim & Abbas, 2021) to reduce the computational construction time of dynamics S-boxes. By supporting sizeable keyspace, the proposed scheme generates an 8x8 s-box in 1ms. The authors’ analysis shows that schemes have a high-level resistance against chosen plaintext and key-related attacks. In Ye & Zhimao (2018), a six-dimensional fractional Lorenz-Duffing chaotic system with an o-shaped path shuffling algorithm was presented to produce a robust high-level S-box. FLDSOP begins by constructing a preliminary S-box using a six-dimensional FLDS. Second, it creates an O-shaped route scrambling method to disrupt the order of the component in the obtained S-box. In Jamal et al. (2019), the chaotic behavior of the modified Tent-Sine map is addressed in this study, and a novel approach for constructing substitution-boxes was offered as a result. To construct robust S-boxes, this novel approach investigates the characteristics of chaos using a TSS map and an algebraic Mobius transformation. The main goal in Khan, Masood & Alghafis (2020) was to provide a safe and resilient algorithm with the least amount of vulnerability possible. They used fractals, Fibonacci, chaotic maps, and compared the outcomes to existing approaches, which revealed unmatched communication security. In Hasanzadeh & Yaghoobi (2020), another fractal-based scheme was proposed in which they use Julia set and three-dimensional chaotic map for image encryption. At first, they encrypted the image with the Julia set by shuffling image layers and then the encryption is done with Julia set by doing same shuffling process. The confusion and diffusion property of algorithm enhanced the robustness of the proposed system.

Azam, Ullah & Hayat (2021) proposed a quick and secure public-key image encryption technique based on elliptic curves. To prevent costly calculations, the sender and receiver in this system precompute a public EC using an efficient search algorithm. This approach scrambles the pixels of a masked image using a dynamic S-box after masking the pixels of a plain text with random integers.

## Proposed cryptosystem

**Figure 1:**
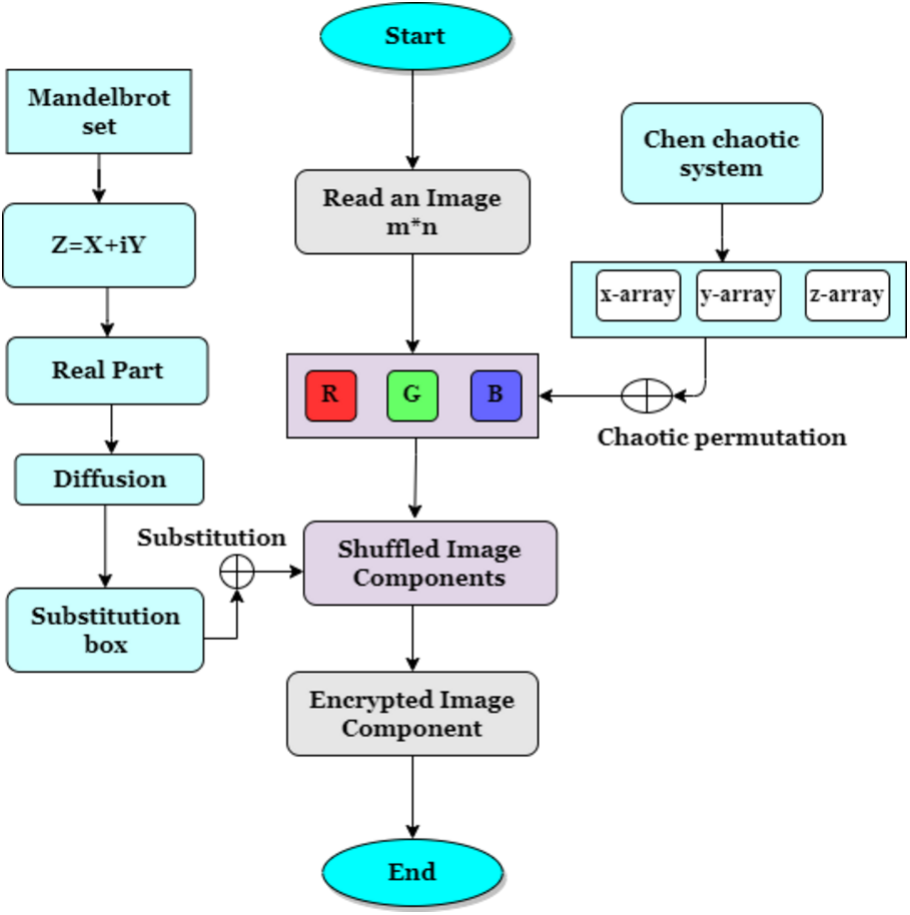
Proposed encryption model.

This section presents the structure of the proposed encryption mechanism first and then describes each algorithm’s working process. The proposed encryption cryptosystem depends on the SP network using Mandelbrot sets and Chen’s chaotic system. The proposed scheme model is shown in Fig. 1. The proposed scheme uses two keys for encryption. The first key is made up of the Mandelbrot set substitution box, creating confusion. The second key is the permutation procedure of the Chen chaotic system, which creates diffusion in image pixels. Both keys increase the robustness of our algorithm to resist brute force attacks. At first, Chen’s chaotic system (key used for permutation) XORed trajectories with image planes and created a nontangible image for adversaries. The one who knows the permutation key can only understand the image information when an image is scrambled with Chen chaotic permutation key. In the second round of image encryption, a new S-box key generated with the Mandelbrot set is used to create confusion in the image. Table 1 below shows the special character used in this article to design the system model.

### Mandelbrot set substitution box

The Substitution box (S-box) is one of the essential non-linear components of the block cipher. The S-box creates confusion in the encryption process due to its non-linearity property. Shannon’s (1949) highly non-linear S-box breaks the relationship between key and ciphertext. We have designed a new structure for the substitution box by implementing Mandelbrot sets. The substitution-box generated by the offered design is highly non-linear and passes all the standard cryptographic analysis. The substitution box is essential for achieving excellent cipher characteristics. Because of its resilience to cryptanalysis, both differential and linear, its significance in any cryptosystem cannot be overstated. When we look at the Mandelbrot set image, we can see the Mandelbrot set in the dark zone. Now to pick any c-value from this dark region for the construction of S-box, we will see that when iterating *x*^2^ + *c*, the orbit of zero does not escape to infinity. The Mandelbrot set in the plane is symmetric with respect to the *x*-axis, and its intersection with the *x*-axis occupies the interval from −2 to 1/4 (Devaney, 2006). The point 0 is located inside the major cardioid, while point −1 is located within the ‘bulb’ to the left of the main cardioid. The *y*-axis of the Mandelbrot set, also known as the imaginary axis, lies between −1 to 1 in the complex plane. As we know, Mandelbrot set shows sensitive behavior at their initial condition, a tinny change will be resultant a different out. To resist brute force attacks, we selected those parameters where the sensitivity of the Mandelbrot set is very high. These are the initial parameters to generate an S-box by fulfilling Mandelbrot set properties. We develop an S-box into two different steps discussed below.

**Table 1:** Comparative analysis of nonlinearity.

Symbols	Meaning	Symbols	Meaning
*λ*	lambda	ẋ	x
⊕	Xor	ẏ	y
∀	For all	ż	z
∈	Belongs to	ḃ	z
*ι*	iota	*ω*	omega
*θ*	theta	Δ	Delta
∩	intersection operator	*ϕ*	phi
⊆	denote a subset	⟶	right arrow
∧	to represent various operations	∪	Union operation

**Figure 2:**
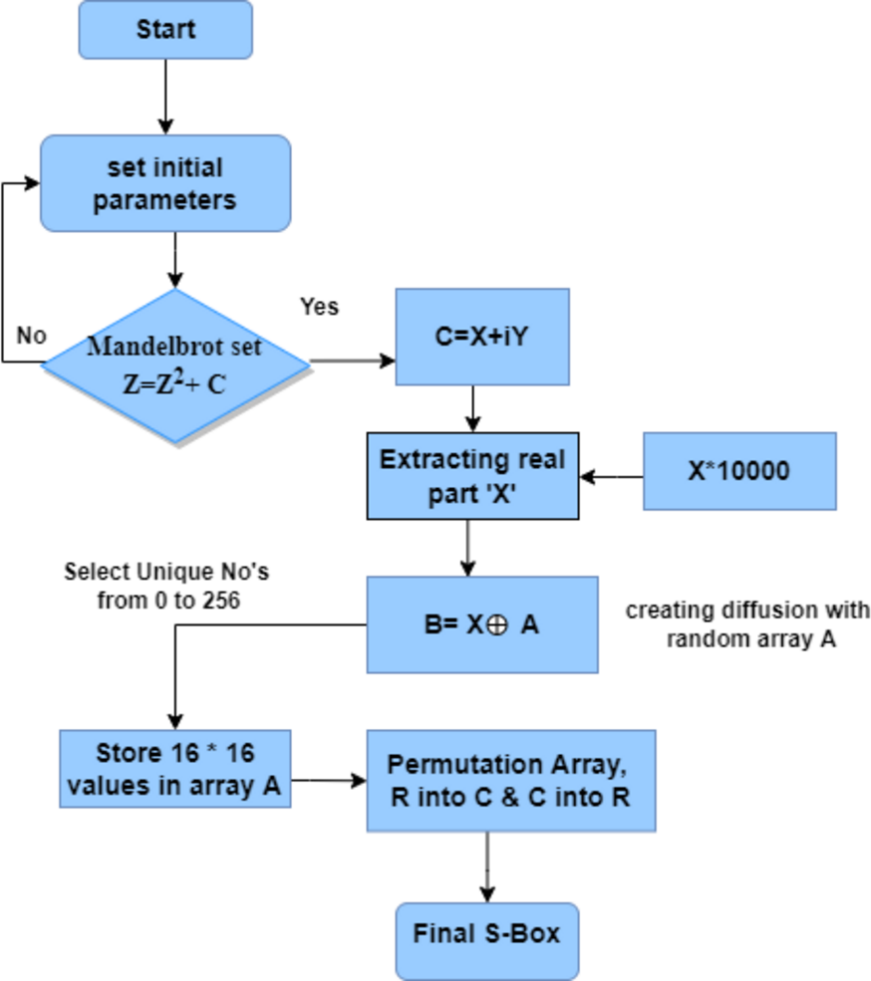
Proposed S-box construction model flowchart.

*Step 1: Initial S-box Generation*:- The choice of an initial S-box is a critical step, and it is the phase that takes us to an outstanding S-box with improved performance. We set the initial parameters manually as *x*_0_ = −1.7, *y*_0_ =0, I = and c we obtained the non-linearity value of 100. We generated the S-box according to the following steps shown in Fig. 2. In our approach, we altered the first S-box to increase the nonlinearity score and.

*Step 2: Final S-box*:- In this step we set the initial parameter manually which satisfied Mandelbrot set conditions as; *x*_0_ = 0.9, *y*_0_ =−0.3, I = −1.45. The output S-box have higher randomness than the previous S-box. To increase randomness, we permuted S-box rows into columns and then columns into rows. From the given S-box, we obtained minimum non-linearity S-box 102.75 and maximum S-box non-linearity 108 and the average of 106.

**Mandelbrot Set S-box Construction Steps:**

**Step 1:** Set initial parameters for the construction of the Mandelbrot set. In our case, we select the initial parameter manually as x, y, and I.

**Step 2:** The output of the Mandelbrot set is in the form of

*Z* = *X* + *iY*

The real part of the Mandelbrot set (X) is utilized to construct the Substitution box.

**Step 3:** The output values (X) are in small numbers so that, the real part of the Mandelbrot set (X) is multiplied with a large random constant such as:

*X* = *X* × 10000

**Step 4:** Next step is the diffusion of random array generated from MATLAB with X obtained from the previous step by:

*B* = *X*⊕*A*

where A is the random array.

**Step 5:** Now unique 256 values are selected using MATLAB command as follows:

S = unique(B,’stable’)

**Step 6:** Reshape the obtained array in 16×16 array matrix and store as S-box.

**Step 7:** Now permute 16*16 matrix value, rows into column and column into rows and stored output as a final S-box.

A = [Array 0, 256 num’s]

P = Randperm(A)

The proposed Mandelbrot set S-box is shown in Table 2.

## Formal Modeling of Mandelbrot set S-box

**Figure 3:**
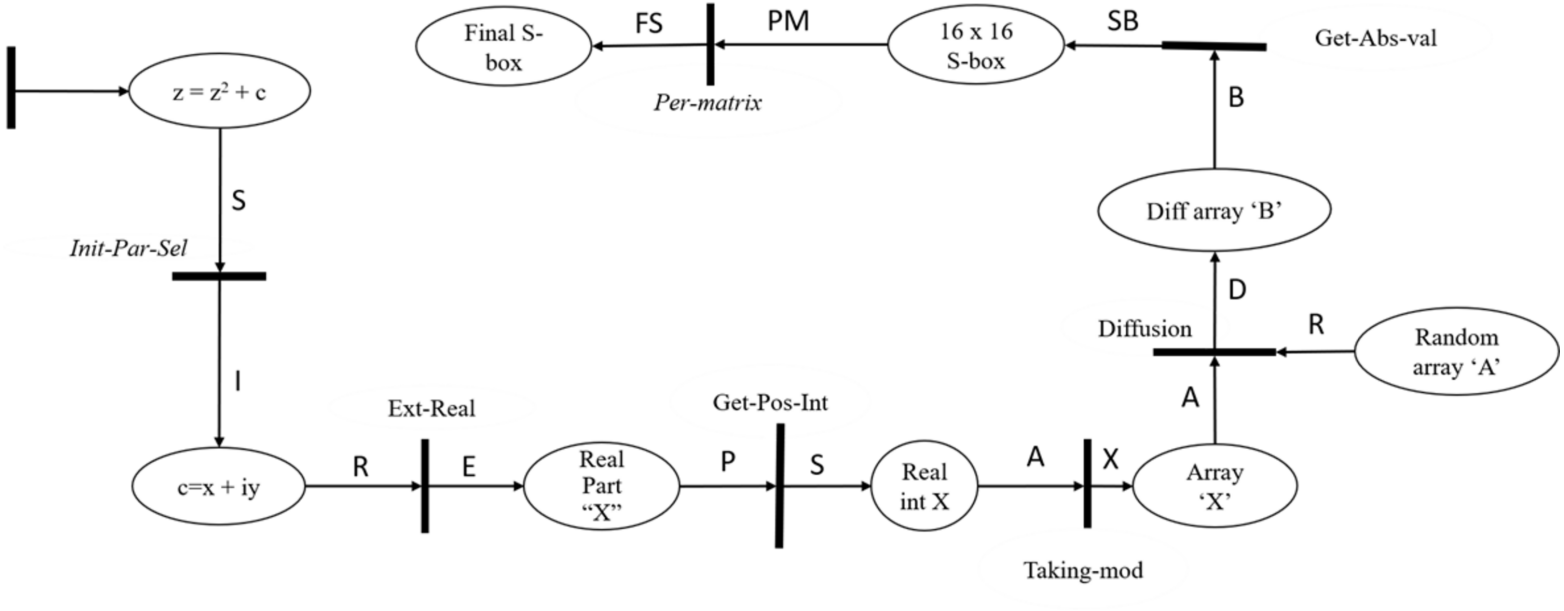
Formal modeling of Mandelbrot set.

The proposed model is designed using high-level Petri nets (HLPN). According to Malik, Khan & Srinivasan (2013), we can utilize HLPN for two reasons: (1) to simulate the suggested systems and (2) to create a mathematical representation to examine the behavior and structural aspects of the proposed model. The advantages of presenting a formal model and analysis of the suggested systems may be summarized as follows: (i) the interconnectedness of the model components and processes, (ii) the fine-grain details of the flow of information among various processes, and (c) how information processing occurs. The SMT is used to verify the proposed S-box construction model; for this reason, the Petri Net models are first translated into SMT with the necessary attributes. Following that, the Z3 solver is used to determine whether the model meets the needed characteristics or not. We utilized HLPN to accomplish the formal definition and modeling of suggested algorithms. Figure 3 shows the HLPN model of the proposed substitution box. HLPN is a set of 7-tuple, N = (P, T, F, ’ R, L, *M*_0_) as discussed in Malik, Khan & Srinivasan (2013) explained below.

**Table 2:** The proposed Mandelbrot S-box.

188	111	117	17	175	20	104	142	171	252	237	191	97	51	66	110	
148	81	211	185	58	189	38	221	155	108	109	22	114	93	153	255	
235	210	122	8	25	150	29	90	57	159	196	182	199	209	95	102	
154	35	123	126	36	34	200	69	162	89	40	193	161	50	14	228	
158	77	241	27	227	133	116	100	45	67	47	112	125	12	30	118	
179	151	178	85	231	249	236	146	119	177	215	78	147	53	33	101	
2	54	92	23	84	251	64	136	203	233	248	28	140	169	247	208	
94	239	224	202	244	87	76	10	72	229	186	183	42	204	198	250	
96	217	172	197	143	74	80	43	18	207	141	201	75	26	157	135	
39	163	205	9	107	13	190	83	174	5	11	60	168	213	99	219	
240	56	79	245	180	226	19	16	167	225	6	128	71	216	206	52	
88	156	70	222	246	55	37	65	242	131	243	139	105	134	103	212	
62	132	63	120	170	138	165	184	160	121	41	176	218	31	149	32	
192	181	49	173	214	59	238	234	4	230	137	7	24	144	220	106	
98	127	194	152	44	86	253	124	1	68	3	82	223	15	195	113	
145	21	129	232	73	254	130	166	48	0	115	164	187	61	46	91

1. P is a set of finite places.
2. T represents a set of finite transitions, such that (P∩*T* = *ϕ*)
3. F denotes the flow relation from place to transition or transition to place, such that $F\subseteq \left( P\times T \right) \cup (T\times P);$
4. *φ* represents the mapping function that maps places to data types, such as *φ*:*P*⟶ Data Types.
5. R represents the rules that map T to logical formulae, such as *R*:*T*⟶ Formula.
6. L denotes the labels mapped on each flow in F, such that *L*:*F*⟶ Label; and 7. *M*_0_ represents the initial state where the flow can be initiated, such that *M*:*P*⟶ Token.

(2) \begin{eqnarray*}R(Init-Par-Set)=\forall s\in S\wedge \forall i\in I{|}\end{eqnarray*}

(3) \begin{eqnarray*}i=s[2]\wedge {S}^{{^{\prime}}}=S\cup \{ i\} \end{eqnarray*}

In this transition, we manually set the initial parameters for constructing the Mandelbrot set Substitution box. By giving the constant values to the initial parameters ‘c’ and ‘i’, where the value of the parameters lies in the Mandelbrot set. The sensitive initial parameters will be selected, creating higher randomness in the proposed system.

(4) \begin{eqnarray*}R(Ext-Real)=\forall r\in R\wedge \forall e\in E{|}\end{eqnarray*}

(5) \begin{eqnarray*}e=Extract(r)\wedge {E}^{{^{\prime}}}=E\cup \{ e\} \end{eqnarray*}

As we know, the complex number is a combination of real and imaginary numbers, a + bi, where ‘a’ is a real part, and ‘bi’ is an imaginary part. For S-box generation, we extracted a real part from iterated complex numbers. *E.g.,* Z = X+ iY is an imaginary number, extract the real part ‘X’ from n iteration and take the modulus 2 of that number. However, values greater than 2 tend towards infinity very fast, which means values greater than 2 are not in the Mandelbrot set.

(6) \begin{eqnarray*}R(Get-Pos-Int)=\forall p\in P\wedge \forall s\in S{|}\end{eqnarray*}

(7) \begin{eqnarray*}s=Product(p,10,000)\wedge {S}^{{^{\prime}}}=S\cup \{ s\} \end{eqnarray*}

In this transition, the outputted values are very small integers to multiply those numbers with a large integer. In our case, we multiply outputted ‘X’ with 10,000 to get a decimal number. Now we have an output array of real numbers; let us say ‘X’ is the outputted array with 0–255 decimal numbers. In this article, we focused on the design algorithm of an 8 × 8 S-box. An 8 × 8 S-box is a number set of 0, 1, 2, …, 255, which is represented by a 16 × 16 matrix *i* = 1, 2,…,16; *j* = 1, 2, …, 16.

(8) \begin{eqnarray*}R(Taking-mod)=\forall a\in A\wedge \forall x\in X{|}\end{eqnarray*}

(9) \begin{eqnarray*}x={|}a{|}\wedge {X}^{{^{\prime}}}=X\cup \{ x\} \end{eqnarray*}

In this transition, we take mod of the values to remain bounded in the Mandelbrot set. The modulus of a complex number is its distance to 0.

(10) \begin{eqnarray*}R(Diffusion)=\forall r\in R\wedge \forall a\in A\wedge \forall d\in D{|}\end{eqnarray*}

(11) \begin{eqnarray*}d=Transform(a,(Rand(r)))\wedge {D}^{{^{\prime}}}=D\cup \{ d\} \end{eqnarray*}

In this transition, we generated a new array, ‘B’, through the nonlinear transformation, the array sequence ‘X’ is transformed with the random sequence ‘A’. The transformation of the random array ‘A’ increases randomness in the array.

(12) \begin{eqnarray*}R(Get-abs-Val)=\forall b\in B\wedge \forall sb\in SB{|}\end{eqnarray*}

(13) \begin{eqnarray*}sb={|}Distinct(b){{|}}_{256}\wedge S{B}^{{^{\prime}}}=SB\cup \{ sb\} \end{eqnarray*}

In this model transition, we selected the unique values from the array ‘B’ and roundoff the values by taking mod 256 and store in a 16 × 16 matrix. The 16 × 16 S-box has values between 0–256.

(14) \begin{eqnarray*}R(Per-matrix)=\forall pm\in PM\wedge \forall fs\in FS{|}\end{eqnarray*}

(15) \begin{eqnarray*}fs=Diffuse(pm)\wedge F{S}^{{^{\prime}}}=FS\cup \{ fs\} \end{eqnarray*}

In this transition to generate a high random S-box, we permutated the rows into columns and columns into rows to create diffusion in the S-box matrix. The diffusion process hides the relationship between plain and cipher images, creating higher resistance against differential and linear attacks. The generated S-box was used for image encryption by creating confusion in image pixels.

### Construction of diffusion key

**Figure 4:**
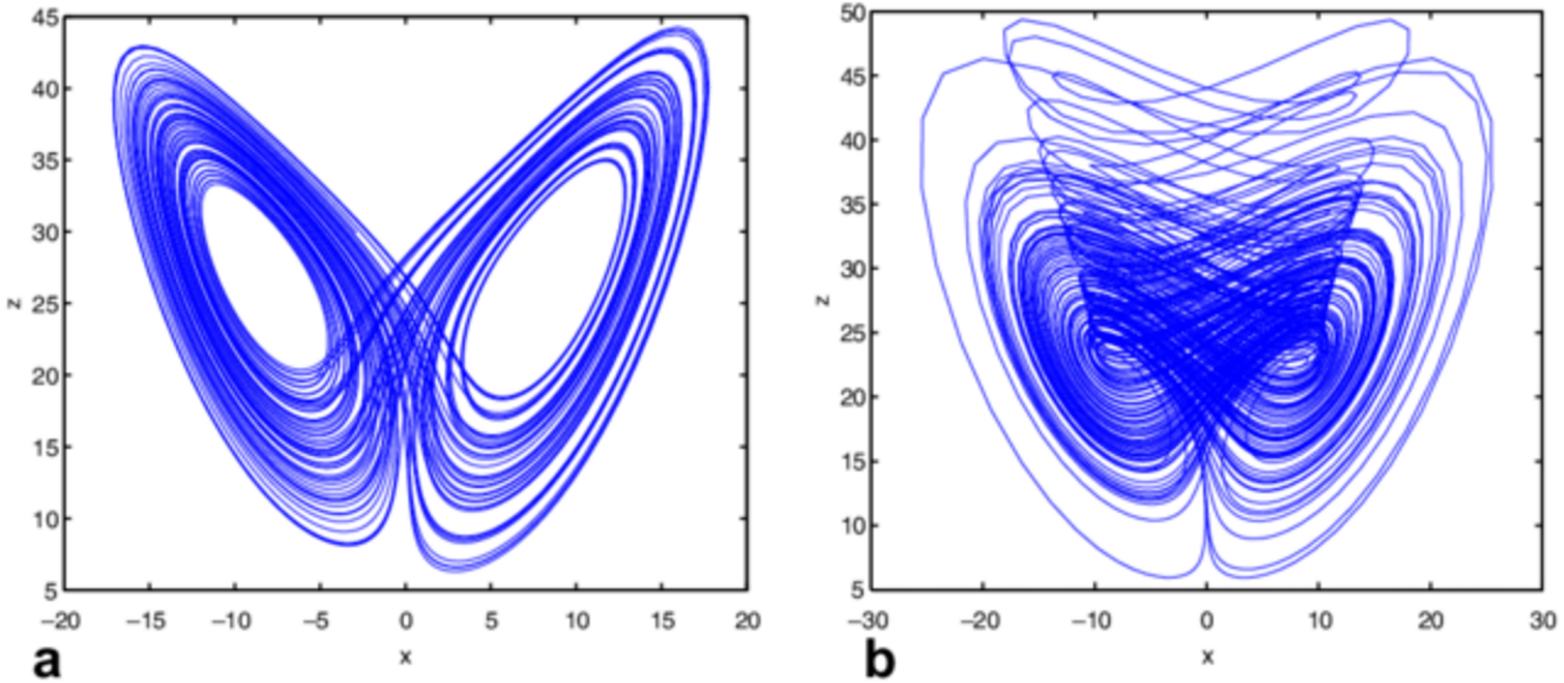
(A–B) Chen’s chaotic system (Chen & Ueta, 1999).

Chaotic systems have been frequently utilized for secure data transmission from past decades. Chaos-based cryptographic algorithms are considered a reliable source of secure encryption due to their sensitivity to the initial condition, Ergodicity, and other chaotic features. The combination of confusion and diffusion gives rise to an SPN. In the proposed encryption process, the diffusion is produced by Chen’s chaotic systems. The diffusion key is constructed by using Chen’s chaotic system with some specific initial conditions and chaotic parameters. Chen’s chaotic system is employed to shuffle the data for diffusion. Chen chaotic has extremely similar equations of Lorenz systems, but there is a topological difference. The parameter c in front of the variable y leads the present system to have a lot of complicated characteristics. Chen’s chaotic system has more complex dynamical characteristics than Lorenz chaotic system. Mathematical general parameter of Chen’s chaotic system (Chen & Ueta, 1999) is defined as:

(16) \begin{eqnarray*} \left\{ \begin{array}{@{}c@{}} \displaystyle dx/dt=a(cy-cx)\\ \displaystyle dy/dt=(c-a)cx+cy-xz\\ \displaystyle dz/dt=-bz+cy \end{array} \right. \end{eqnarray*}

where a, b, and c are parameters. After executing the diffusion and confusion process, the encrypted data is passed through cryptographic standards to measure its strength. Chen’s chaotic attractor is shown in Fig. 4 (Cassal-Quiroga & Campos-Cantón, 2020).

### Image encryption process

The following are the steps in the proposed encryption scheme:

**Step 1:** Insert an image m×n×3 as input of encryption scheme and split each channel of an image as red, green, blue.

**Step 2:** Initialize Chen’s chaotic map using chaotic sequence and initial parameters.

**Step 3:** The trajectories of Chen’s chaotic map are stored as x, y, and z arrays from Eq. (16).

**Step 4:** Each trajectory is utilized to permute channels of an image.

**Step 5:** After scrambling Chen’s chaotic trajectories with the Image plane, a new scrambled image is generated.

**Step 6:** The Mandelbrot substitution box is implemented on an obtained image from step 5.

**Step 7:** At the end, the obtained resulting layers are compiled as cipher images.

## Performance Analysis and Results

This section describes the proposed encryption scheme’s evaluations and simulation results. The performance study shows that the encryption approach encrypts the original image and changes all its information, increasing unpredictability. An ideal encryption method generates a cipher image with high unpredictability in the encrypted pixels of the image. The simulation and analysis work was performed at MATLAB R2018a, core i5,8GB RAM win 10.

### Non linearity

The main objective of S-box is to aid the non-linear transformation of unique information into encoded data non-linearity defines the gap between function and all affine functions. The number of bits in a Boolean truth table may represent how it must be modified to gain the nearest affine function. The non-linearity score of a cryptographic function can be used to determine its susceptibility to linear attacks. The Walsh spectrum can represent the Boolean function non-linearity f(x):

(17) \begin{eqnarray*}{N}_{f}={2}^{m-1}\dot {(1-{2}^{-m})}ma{x}_{\omega \in GF({2}^{m})}{|}{S}_{f}(\omega ){|}\end{eqnarray*}

And Walsh spectrum is

**Figure 5:**
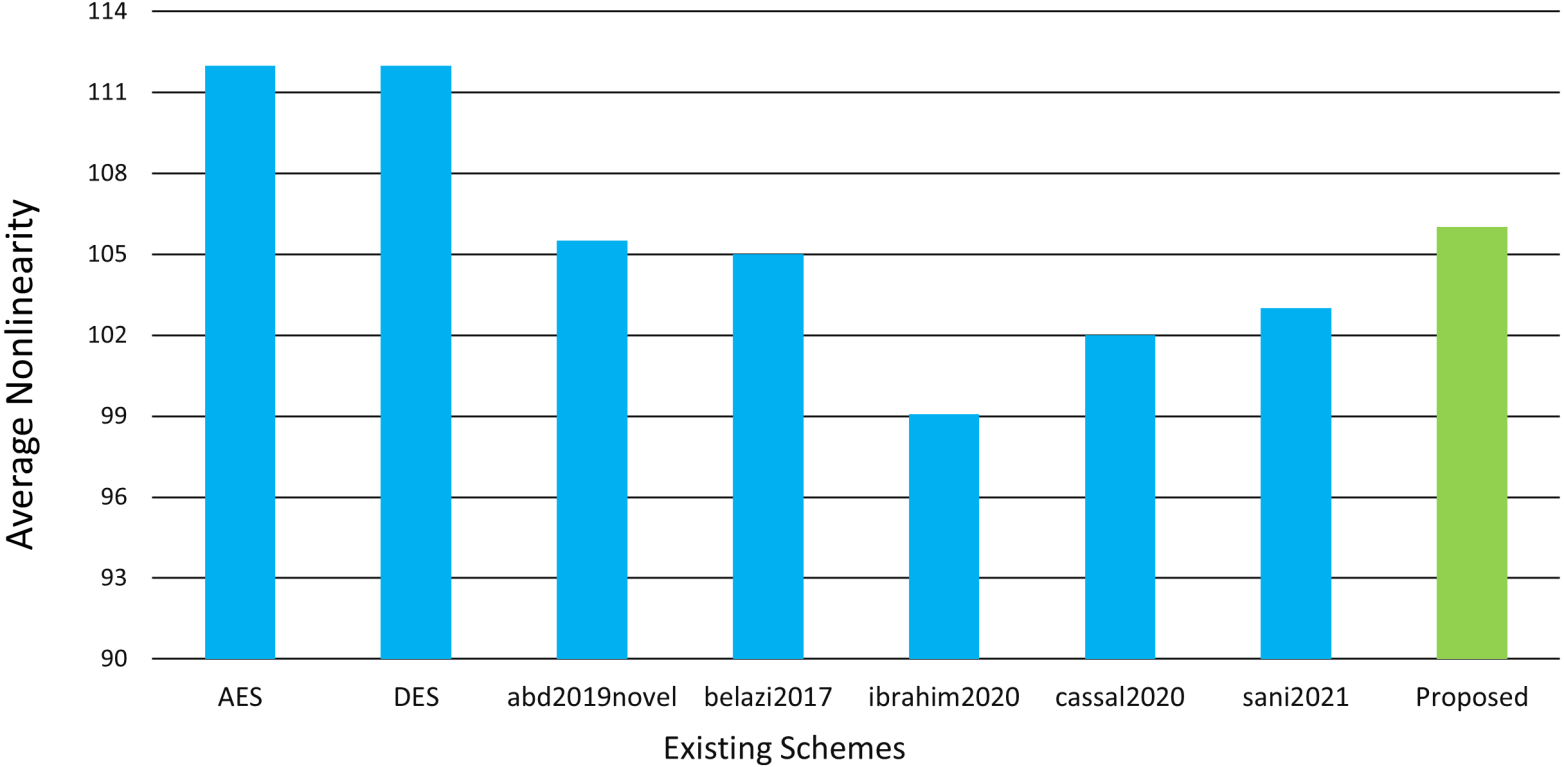
Nonlinearity comparison chart.

(18) \begin{eqnarray*}{S}_{f}(\omega )=\sum _{x\in GF({2}^{m})}[-1]^{f(x)}\oplus x\dot {\omega }\end{eqnarray*}

whereas *ω* belongs to 2^*m*^. High non-linearity values are obtained using the methodology utilized in this article. Linear approximation and affine attacks are examples of cyber-attacks that highlight the need for S-boxes with high non-linearity values. As a result, the significant non-linearity of our suggested S-box aids in providing great confusion as well as great resilience to these attacks. The resulting values from the highest nonlinear S-box max, min, and average scores are 108, 104, 106, respectively. Table 3 and Fig. 5 compare the proposed S-box to existing systems in terms of non-linearity.

**Table 3:** Comparative analysis of nonlinearity.

algo	Max	Min	Avg
AES	112	112	112
APA	112	112	112
Grey	112	112	112
Skipjack	108	104	105.25
Al Solami et al. (2018)	110	106	108
Abd EL-Latif, Abd-El-Atty & Venegas-Andraca (2019)	108.2	104	106.2
Belazi & Abd El-Latif (2017)	110	102	105.5
Liu, Zhang & Wang (2018)	108	102	104.5
Vaicekauskas, Kazlauskas & Smaliukas (2016)	108	98	102.5
Silva-García et al. (2018)	106	100	103
Hayat & Azam (2019)	102	56	92.44
Ibrahim & Alharbi (2020)	106	82	99.07
Cassal-Quiroga & Campos-Cantón (2020)	108	96	102
Ye & Zhimao (2018)	106.2	104	105.2
Özkaynak, Çelik & Özer (2017)	107	101	104.5
Khan, Shah & Batool (2016)	106	84	100
Sani, Behnia & Akhshani (2021)	106	98	103.7
**Proposed**	108	104	106

### Strict avalanche criterion

Webster & Tavares (1985) presented the concept of exacting strict avalanche effect (SAC), which is the speculation of culmination and torrential slide impact in the year 1985. SAC is used to check the confusion capability. It is especially beneficial in Shannon-based encryption, where a slight modification might result in a 50 % difference in the output bits. Table 4 shows the dependence matrix for S-box.

**Table 4:** Proposed S-box SAC.

0.484375	0.578125	0.531250	0.500000	0.484375	0.515625	0.515625	0.531250
0.515625	0.515625	0.531250	0.484375	0.406250	0.531250	0.609375	0.484375
0.578125	0.453125	0.546875	0.468750	0.531250	0.484375	0.515625	0.421875
0.562500	0.515625	0.500000	0.500000	0.468750	0.468750	0.500000	0.500000
0.625000	0.484375	0.484375	0.437500	0.453125	0.468750	0.437500	0.609375
0.531250	0.484375	0.453125	0.500000	0.531250	0.468750	0.468750	0.500000
0.484375	0.500000	0.515625	0.453125	0.453125	0.515625	0.421875	0.500000
0.500000	0.531250	0.421875	0.531250	0.468750	0.484375	0.500000	0.437500

Every unit of the resultant table shows that the SAC value is close to 0.5, which is acceptable. The proposed construction S-box accomplishes the least, most extreme, and ordinary qualities for SAC is 0.406250, 0.625000, and 0.498291 separately, and the difference is 0.044987.

### Bit independence criteria

Bit independent criteria are used to maintain the capacity of disarray work in replacement boxes. Webster & Tavares (1985) first defined this measurable property, *e.g.*, for a set of specific torrential slide vectors, in general, the torrential slide factors must be pairwise free. For proposed S-boxes, the average BIC non-linearity matrix is 106, which is a significant value. Furthermore, the BIC-SAC matrix’s average against our S-box is 0.50021, which is quite near 0.5. Table 5 presents the BIC, and Table 6 presents the SAC of BIC. The findings provided in Table 7 demonstrate that our suggested S-box is very desired based on SAC and BIC criteria.

**Table 5:** Bit independence criteria.

—-	104	108	104	104	104	104	104
104	—	98	104	98	108	108	102
108	98 —	104	100	108	108	104
104	104	104	—	108	108	104	96
104	98	100	106	—	104	102	102
104	106	108	106	104	—	106	102
104	108	108	104	104	106	—	102
104	102	104	96	102	102	102	—

**Table 6:** SAC of BIC.

—	0.496094	0.509766	0.482422	0.527344	0.519531	0.505859	0.533203
0.496094	—	0.484375	0.498047	0.505859	0.482422	0.501953	0.505859
0.509766	0.484375	—	0.498047	0.490234	0.480469	0.501953	0.496094
0.482422	0.498047	0.498047	—	0.511719	0.494141	0.488281	0.490234
0.527344	0.505859	0.490234	0.511719	—	0.505859	0.496094	0.498047
0.519531	0.482422	0.480469	0.494141	0.505859	—	0.501953	0.500000
0.505859	0.501953	0.501953	0.488281	0.496094	0.501953	—	0.500000
0.533203	0.505859	0.496094	0.490234	0.498047	0.500000	0.500000	—

**Table 7:** Proposed S-box BIC-NL and BIC-SAC comparison.

S-box	BIC-NL	BIC-SAC
AES	112	0.5046
APA	112	0.4997
Gray	112	0.5026
Al Solami et al. (2018)	104	0.5006
Abd EL-Latif, Abd-El-Atty & Venegas-Andraca (2019)	103.7	0.5065
Belazi & Abd El-Latif (2017)	103.78	0.4970
Liu, Zhang & Wang (2018)	104.6	0.508
Cassal-Quiroga & Campos-Cantón (2020)	101.75	0.5066
Ibrahim & Alharbi (2020)	99.9	0.4868
**Proposed**	106	0.50021

After that exacting torrential slide standard on BIC, SAC was applied, and the outcome is given in Table 7. The standard worth of SAC of BIC is 0.50021, which demonstrates the power of the presented substitution box.

### Differential approximation probability

**Figure 6:**
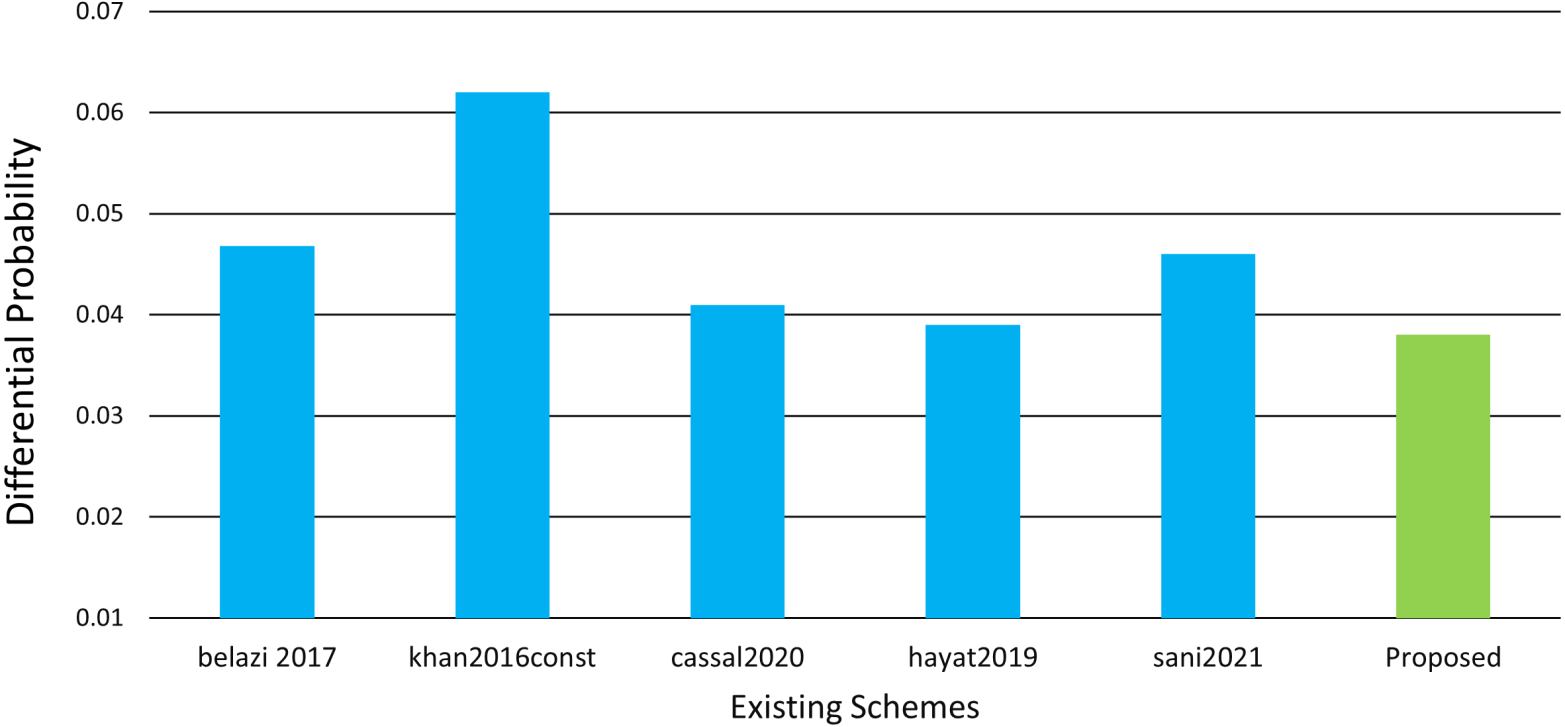
Differential probability comparison with literature.

The differential approximation probability for an S-box should demonstrate differential uniformity under ideal conditions. An S-box is considered as strong as much smaller DP values. The proposed S-box differential probability is shown in Table 8 and Fig. 6. According to Eq. (19) (Dimitrov, 2020).

(19) \begin{eqnarray*}DAP(\lambda x\rightarrow \lambda y)= \frac{{|}x\in X{|}(Sx)\oplus (x\oplus \lambda x\oplus =\lambda y)}{{2}^{m}} \end{eqnarray*}

**Table 8:** DP and LP of proposed S-box.

**S-box**	**DP**	**LP**
Liu, Zhang & Wang (2018)	0.047	0.125
Belazi & Abd El-Latif (2017)	0.0468	0.1250
Silva-García et al. (2018)	0.03906	0.1391
Vaicekauskas, Kazlauskas & Smaliukas (2016)	0.062	0.141
Hayat & Azam (2019)	0.0391	0.1484
Ibrahim & Alharbi (2020)	0.0391	0.1250
Özkaynak, Çelik & Özer (2017)	0.396	0.140
Khan, Shah & Batool (2016)	0.624	0.179
Sani, Behnia & Akhshani (2021)	0.046	0.1298
**Proposed**	0.0380	0.1328

*λx* is an input and *λy* is output differential, x is the set of all possible inputs, and 2^*m*^ isthe number of elements.

### Linear approximation probability

The linear approximation probability is often used to assess an event’s mismatch. This number helps determine the highest value of the event’s mismatch, accordingly, i and j are used to determine the parity of input and output bits. The linear probability can be calculated through the adapted Eq. (20) (Dimitrov, 2020) below.

(20) \begin{eqnarray*}L{P}_{f}=ma{x}_{i,j}\not = \frac{x\in X{|}x.i=S(x)j}{{2}^{m}} = \frac{1}{2} \end{eqnarray*}

where i is the input mask value and j is the output mask value, X is a set of 2^*m*^ elements that contains all x input values. An S-box is considered as strong as smaller the values of linear approximation probability. In the proposed S-box case, the maximum LP is 0.13281, as shown in Table 8.

## Statistical analysis

The connection between the plain image and the cipher image is defined by statistical analysis. These sorts of analyses are carried out to crack various types of cryptosystems. Image encryption statistical analysis is extremely crucial. A strong image encryption algorithm must be resilient to any statistical attack. Image histogram analysis and the correlation of neighboring pixels are two critical statistic measurements of image encryption algorithms. Furthermore, in this section, we performed further security analyses such as Differential analysis and NIST 800-22 analysis.

### Histogram analysis

**Figure 7:**
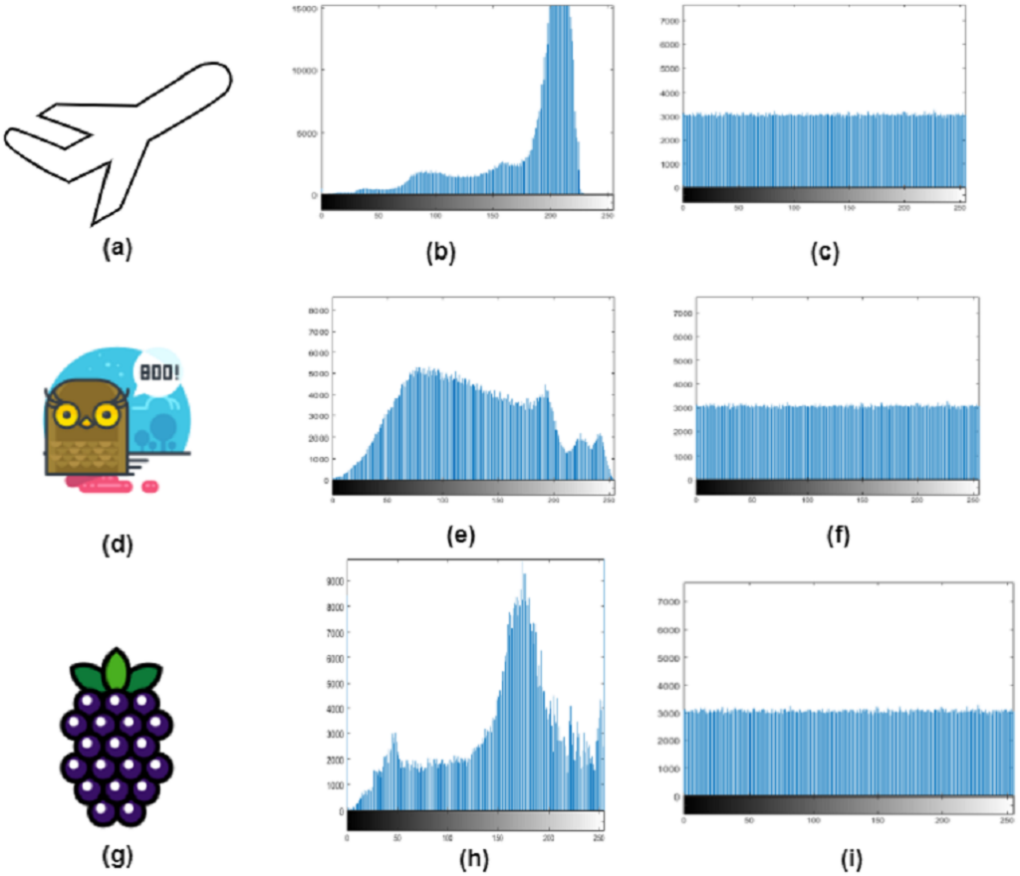
Original image (A, D, G), and histogram analysis of the original image (B, E, H), and cipher images histogram (C, F, I).

**Figure 8:**
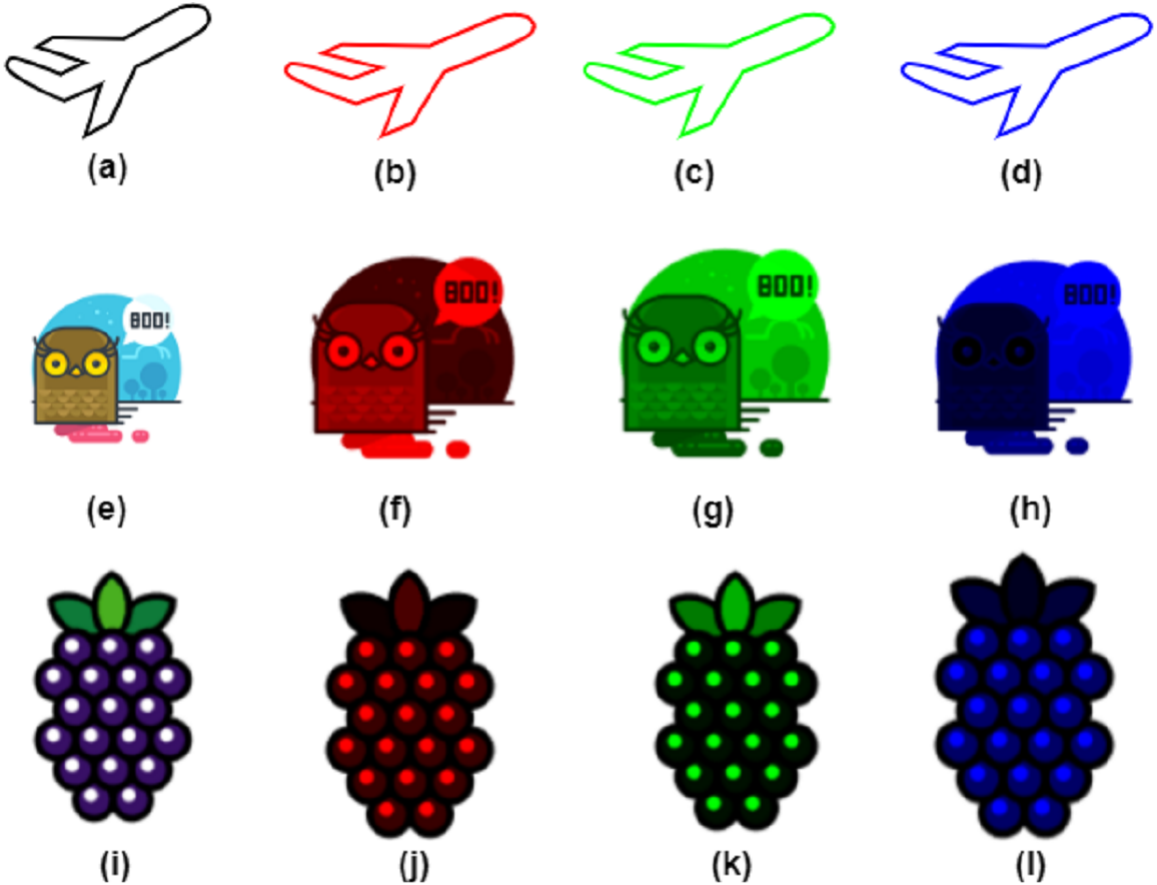
RGB channel separation. (B, F, J) red channel, (C, G, K) green channel, (D, H, L) blue channel of airplane, owl and fruits test images.

**Figure 9:**
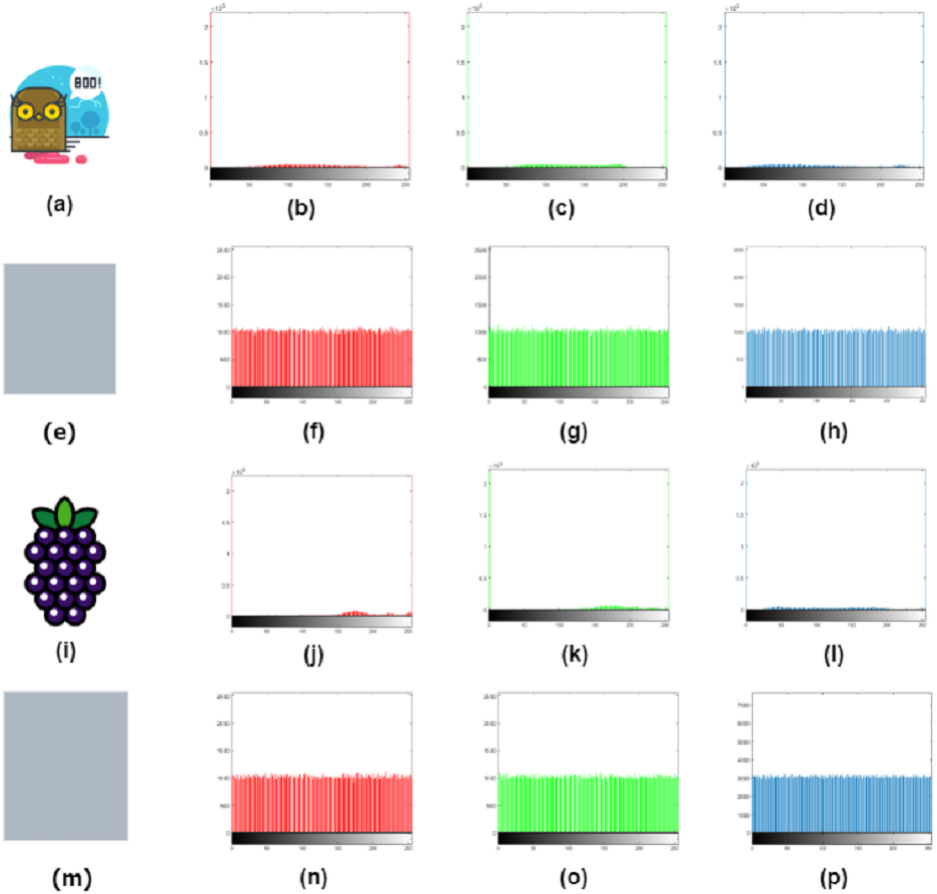
Histogram of RGB channel of owl (B, C, D); encrypted image channel histogram (F, G, H), histogram of RGB channel of fruits (J, K, L), encrypted image channel histogram (N, O, P).

Histogram analysis is the analysis of graphical values for image information. It is used to validate the pixel distribution values for the cipher image. A suitable type of image encryption has the same frequency of grey scales, which indicates a uniform distribution. The cipher image’s histogram shows a balanced distribution of pixels. This demonstrates that it is challenging for attackers to obtain useful statistical information from the encrypted image. Although the pixel values of the encrypted image do not have a simple regularity, the attacker cannot extract the original image by a raw force analysis of the cipher. The histogram analysis for the original and cipher images is shown in Fig. 7. The encryption process is comprised of the permutation stage where the Chen chaotic trajectories would XORed with image channels to create diffusion. The comparison of the RGB channels of the original and cipher image test the encryption efficiency. The RGB channel of original test images are shown in Fig. 8 and the RGB channel-wise histogram analysis of test images is shown in Fig. 9.

### Coefficient analysis

**Figure 10:**
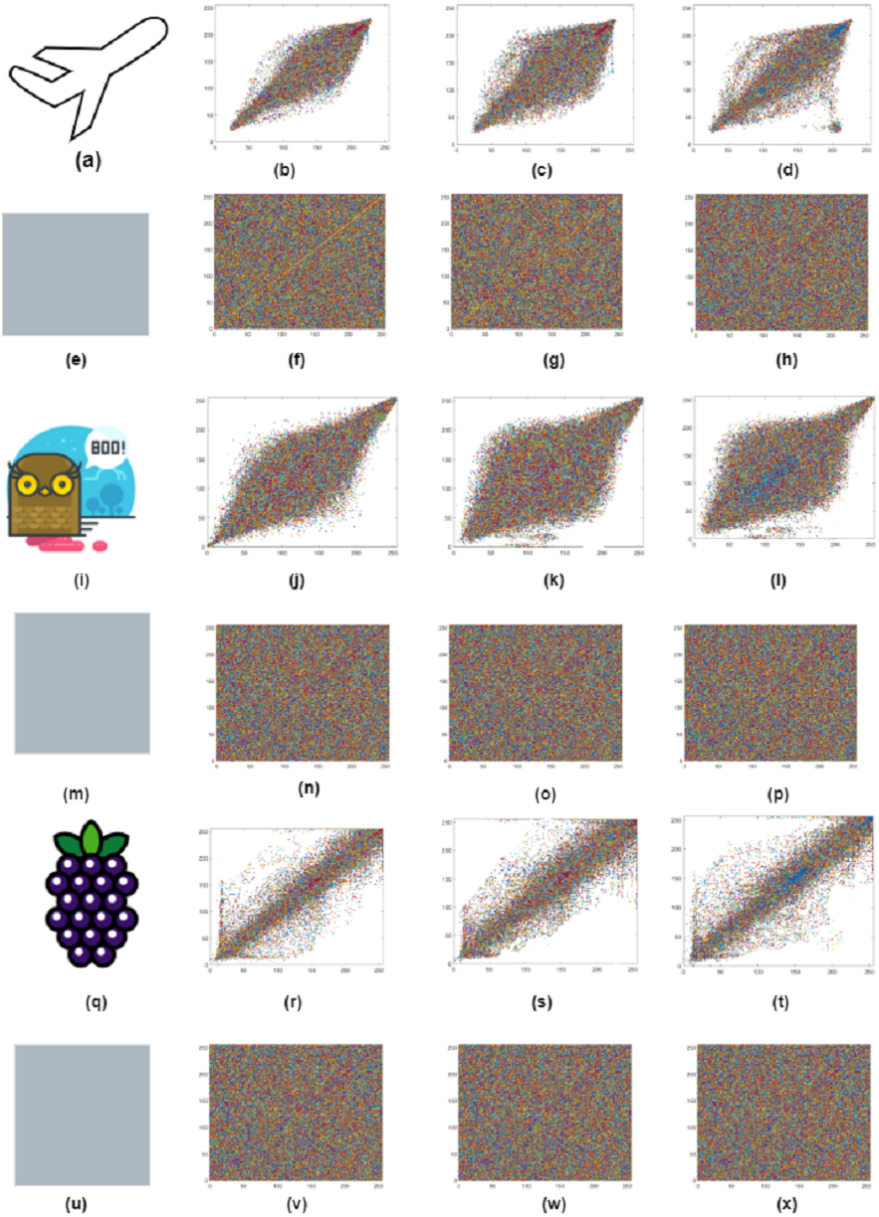
(A–X) Correlation coefficient of original and cipher images airplane, owl and fruits.

**Figure 11:**
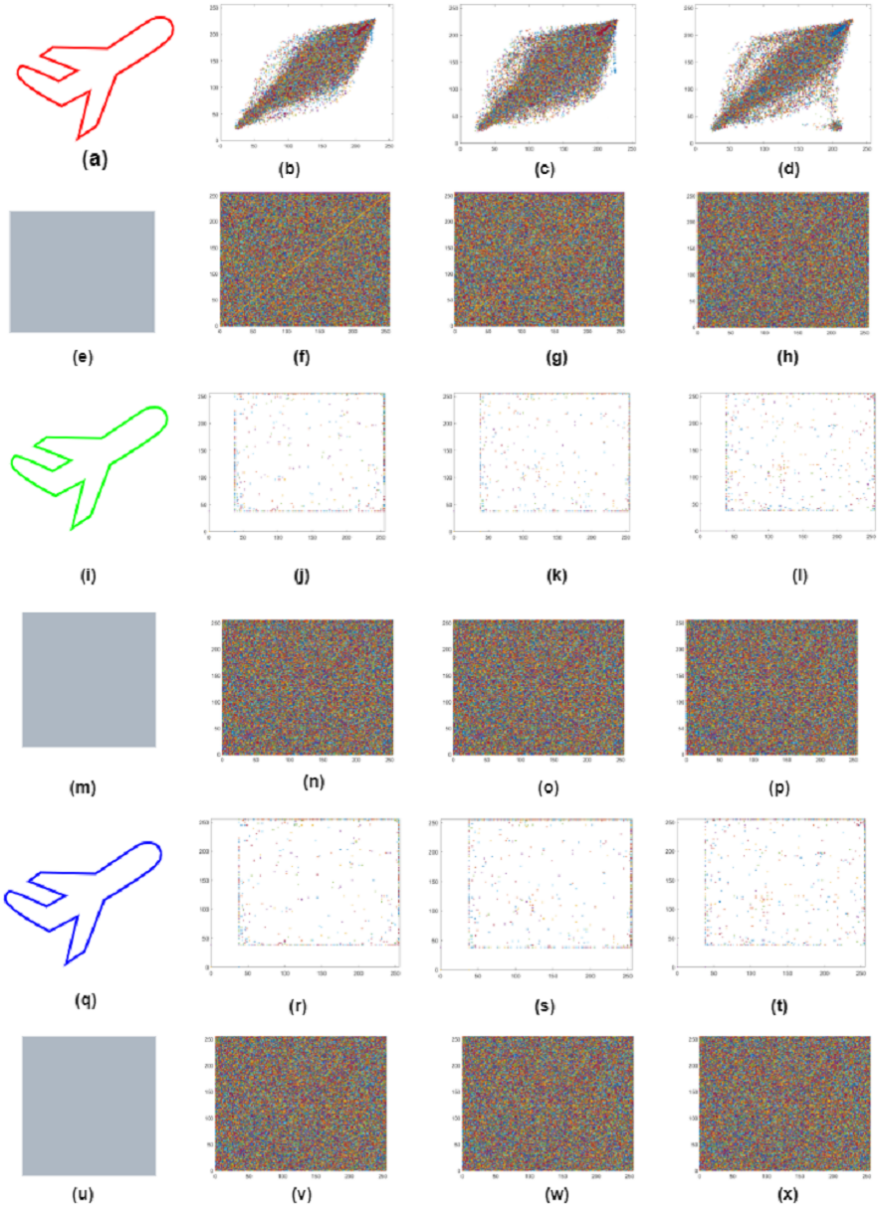
Layer wise correlation of airplane original and cipher image. (B, F, J, N, R, V) red, green and blue Horizontal plane correlation; (C, G, K, O, S, W) red, green and blue diagonal plane correlation; (D, H, L, P, T, X) shows red, green and blue vertical plane correlation.

The correlation will visually represent the difference between the plain and cipher images than the histogram and data entropy. The association between neighboring pixels is reasonably high in plain images, and the attacker can use the correlation between neighboring pixels to gain useful information. Therefore, the similarity between the neighboring pixels of the encrypted image is closer to 0 after image encryption, suggesting that the pixel distribution is random. Attackers carry out a statistical attack using the input image’s correlation values. The encryption algorithm is thus necessary to decrease the association between adjacent pixels of cipher images. The correlation coefficient of the original and cipher image of the airplane, owl, and fruits is shown in Fig. 10 and in Table 9. The channel-wise correlation coefficient of the original and cipher Airplane image is shown in Fig. 11. The correlation coefficient can be represented mathematically as an adapted Eqs. (21) to 23 (Şengel, Aydın & Sertbaş, 2020) below.

(21) \begin{eqnarray*}S= \frac{\mathbf{cov}~(\mathbf{x1},~\mathbf{y1})}{\alpha x~\cdot ~\alpha y} \end{eqnarray*}

**Table 9:** Correlation coefficient.

Test image	Channels	Original image			Cipher image		
		**Horizontal**	**Diagonal**	**Vertical**	**Horizontal**	**Diagonal**	**Vertical**
Baboon	RGB	0.9231	0.8543	0.8660	0.0001	0.0041	0.0013
	Red	0.9459	0.9054	0.9399	0.0001	0.0041	0.0013
	Green	0.9897	0.9841	0.9935	0.0005	−0.0008	0.0012
	Blue	0.9897	0.9841	0.9935	−0.0034	0.0025	−0.0021
Fruits	RGB	0.9726	0.9523	0.9728	0.006	−0.00021	0.0015
	Red	0.9279	0.8831	0.9478	0.0013	0.0009	−0.0043
	Green	0.9897	0.9841	0.9935	0.0009	−0.0009	−0.0008
	Blue	0.9897	0.9841	0.9935	−0.0007	−0.0029	0.0020
House	RGB	0.9536	0.9224	0.9579	0.6918	0.5803	0.6921
	Red	0.9464	0.9077	0.9555	0.0006	−0.0021	0.0015
	Green	0.9897	0.9841	0.9935	−0.0017	−0.0026	0.0006
	Blue	0.9897	0.9841	0.9935	−0.0004	0.9947	0.0014
Airplane	RGB	0.9726	0.9523	0.9728	0.6911	0.5804	0.6928
	Red	0.9293	0.8847	0.9488	−0.0027	0.0006	0.0005
	Green	0.9897	0.9841	0.9935	0.0001	−0.0001	0.0011
	Blue	0.9897	0.9841	0.9935	0.0029	−0.0003	−0.0010

where, $ax=\sqrt{var}{x}_{1}$ and $ay=\sqrt{var}{y}_{1}$

(22) \begin{eqnarray*}Var(x1)= \frac{\mathbf{1}}{n} \sum _{i=\mathbf{1}}^{n}(xi-E(x1))^{\mathbf{2}}\end{eqnarray*}

(23) \begin{eqnarray*}Cov(x1,y1)= \frac{\mathbf{1}}{n} \sum _{i=\mathbf{1}}^{n}(xi-E(x\mathbf{1}))(yi-~E(y\mathbf{1}))\end{eqnarray*}

where x1 and y1 are the original and encrypted image pixels, respectively, and m x n denotes the image’s total dimension. The original and encrypted airplane correlation coefficient can be seen in Fig. 9.

### Entropy analysis

Entropy is the level of uncertainty of pixels in images. Typically, to characterize the intensity of randomness, we use information entropy. This research is a mathematical analysis of the condition. The encrypted image entropy analysis shows its instability, and the resulting values should be like 8 since all the numbers have the same probability. As a result, the effective cryptosystem entropy should be 8, indicating that all states of information occurred an equal number of times and securing the image against retrieving by the attacker.

(24) \begin{eqnarray*}H=\sum _{n=0}^{n=255}{P}_{n}{\log \nolimits }_{2}{P}_{n}\end{eqnarray*}

where Pn is the frequency of events for pixels of the quality n, local entropy best represents the unpredictability of pixel values in the image. Allow the image to be partitioned into small blocks of equal size that do not overlap, then calculate the entropy of each block of data. The image’s local entropy is defined as the average block entropy values. The information entropy of the channel cipher image shows that most of the values are near to 8, as shown in Table 10.

**Table 10:** Information entropy.

**Images**	Original image planes entropy			Cipher image planes entropy			Entropy	
	**Red**	**Green**	**Blue**	**Red**	**Green**	**Blue**	**Original image**	**Cipher image**
**Airplane**	2.94380	2.9562	2.8377	7.9994	7.9995	7.9994	6.6639	7.9998
**Baboon**	3.1511	3.1025	3.1609	7.9993	7.9993	7.9993	7.7624	7.9998
**Fruits**	2.9169	3.0223	3.1340	7.9992	7.9993	7.9993	7.6319	7.9998
**House**	3.0905	3.0430	3.0902	7.9993	7.9991	7.9992	7.4858	7.9998
**Peppers**	3.0714	3.0225	2.9282	7.9994	7.9993	7.9994	7.6698	7.9998
**Lena**	3.1020	3.0088	2.9491	7.9993	7.9994	7.9993	7.4767	7.9998

### Differential attack analysis

In a differential attack, an attacker changes the slight pixel value of an encoded image and then generates an output image to extract essential information from an image. The phenomenon of differential attack is as simple as using the changed pixel value image and original cipher image and measuring the contrast between them. In this manner, most attackers will break cryptosystems by determining the difference between two encoded images. This approach is known as a differential attack. The suggested algorithm must be susceptible to the secret key and the plain text for a safe encryption strategy. Any minor change in the secret key or the plain text causes the ciphertext to change completely. A number of pixels change rate (NPCR), and Unified average changing intensity (UACI) check the resistance against differential attack. Examination of arithmetic results of NPCR esteems concerning distinctive image layer parts are indicated in Table 11.

(25) \begin{eqnarray*}NPCR({I}_{1},{I}_{2})= \frac{\sum x,yC(x,y)}{w\ast H} \times 100\end{eqnarray*}

and

(26) \begin{eqnarray*}UACI({I}_{1},{I}_{2})= \frac{1}{H\ast W} \left[ \begin{array}{@{}c@{}} \displaystyle \sum xy \frac{ \left\vert {I}_{1}(x,y)-{I}_{2}(x,Y) \right\vert }{255} \\ \displaystyle \end{array} \right] \end{eqnarray*}

where, *I*_1_(*x*, *y*) and *I*_2_(*x*, *y*) are two images with one pixel difference.H * W is the size of image. C(x,Y) is defined as:

(27) \begin{eqnarray*} \left\{ \begin{array}{@{}c@{}} \displaystyle C(x,y)=1 if {I}_{1}(x,y)={I}_{2}(x,y)\\ \displaystyle C(x,y)=0 otherwise \end{array} \right. \end{eqnarray*}

### NIST analysis

The NIST 800-22 test is used to evaluate the randomness characteristic of encryption algorithms. Some qualities, such as extended time, high complication, uniform distribution, and productivity, are used to measure cryptosystem security. We choose several cipher images for the NIST test and compute their *P* values according to Karell-Albo et al. (2020), which should be in the range [0,1]. To confirm the randomization test, we chose three distinct photos from Table 12 (Lena, peppers, and baboon).

**Table 11:** Differential attack results, NPCR and UACI values.

**Images**	NPCR			UACI		
	**Red**	**Green**	**Blue**	**Red**	**Green**	**Blue**
**Airplane**	99.59	33.41	99.61	99.62	33.50	33.39
**Baboon**	99.64	99.60	99.63	33.45	33.51	33.45
**Peppers**	99.61	99.62	99.62	33.41	33.45	33.48
**Fruits**	99.61	99.60	99.62	33.57	33.56	33.44
**House**	99.59	99.60	99.59	33.42	33.42	33.46
**Sailboat**	99.62	99.60	99.61	33.49	33.47	33.51

**Table 12:** NIST measures for different standard color images.

**Test**	*P*- Values		
	** Pepper**	**Baboon**	**Status**
**Frequency**	0.0881	0.8248	Qualify
**Block frequency**	0.0941	0.6331	Qualify
**Serial 1**	0.5501	0.7011	Qualify
**Serial 2**	0.5589	0.1374	Qualify
**Approximate entropy**	0.1430	0.1574	Qualify
**Non-overlapping template**	0.4677	0.8245	Qualify
**Runs**	0.3689	0.5005	Qualify
**Longest run**	0.0753	0.0753	Qualify
**Rank**	0.2919	0.2919	Qualify
**Cumulative sums**	0.1850	0.4022	Qualify
**Overlapping template**	0.8165	0.8596	Qualify
**Universal**	0.9986	0.6669	Qualify

### Key sensitive test

Assume a 16-character cipher key is used in the key sensitivity test. This signifies that the length of the cipher key is 128 bits. The steps below are used to conduct a typical key sensitivity test.

1. To begin, a 512 × 512 image is encrypted using the cipher key “abc123def456gh78”.

2. The least significant bit of the key is then modifying, and the original key becomes, say “abc123def456gh79”.

3. Finally, the two images are encrypted with old and modified cipher keys, and then both images will compare. Therefore, the image encrypted with the cipher key “abc123def456gh78” differs from the image encrypted by “abc123def456gh79” in terms of pixel grey-scale values by 99.61%, even though the difference between both cipher key values is just one bit.

### Mean Square Error (MSE)

The MSE represents the difference between the original and encrypted images. For improved performance between two distinct images, this difference must be quite large. MSE = (1)/(MN)(original image–encrypted image) The MSE measures an estimator’s quality; it is always non-negative, and numbers closer to zero are preferable. The MSE is the estimator’s variance and is measured in the same units evaluated by the quality square. The MSE values for test images are shown in Table 13.

**Table 13:** Mean Square Error (MSE).

**Images**	Values
**Airplane**	427.6257
**Baboon**	390.3983
**Fruits**	419.768
**House**	401.043

## Proposed Scheme comparative analysis

This section compares our scheme to those already published in the literature. The correlation coefficient values and the entropy findings are the significant comparisons we have made here. Table 14 shows a quick correlation coefficient comparison of various common RGB images. The comparison table demonstrates that the correlation values for specific encrypted images are near to or less to zero. These nominal values indicate no relationship between the cipher’s surrounding pixels. Furthermore, by comparing our proposed encryption technique to existing systems, we can demonstrate that it fulfills the requirements of a modest encryption algorithm. The Information entropy shows the high unpredictability between encrypted image pixels. We can demonstrate that our suggested encryption approach creates cipher images with entropy near the ideal value by comparing common encrypted images with the existing system. Table 15 shows a comparison of information entropy.

**Table 14:** Correlation comparison of proposed scheme with existing.

**Images**	Channels	Proposed method			Munir et al. (2020)		
		**Horizontal**	**Diagonal**	**Vertical**	**Horizontal**	**Diagonal**	**Vertical**
Baboon	R	0.0001	0.0041	0.0013	−0.0038	0.0005	0.0010
	G	0.0005	−0.0008	0.0012	0.0006	−0.0002	0.0015
	B	−0.0034	0.0025	−0.0021	−0.0008	0.0005	−0.0019
Fruits	R	0.0013	0.0009	−0.0043	0.0013	−0.0001	0.0026
	G	0.0009	−0.0009	−0.0008	0.0034	−0.0026	0.0020
	B	−0.0007	−0.0029	−0.0020	−0.0000	0.0000	−0.0007
House	R	0.0006	−0.0021	0.00015	−0.0042	−0.0042	0.0041
	G	−0.0017	−0.0026	0.0006	−0.0006	0.0017	0.0021
	B	0.0004	0.9947	0.0014	0.0003	−0.0019	−0.0022
House	R	−0.0027	0.0006	0.0005	0.0011	0.0030	−0.30013
	G	0.0001	−0.0001	0.0011	0.0030	−0.0014	0.0016
	B	0.0029	−0.0003	−0.0010	−0.0017	−0.0021	−0.0004

**Table 15:** Information entropy comparison.

**Images**	Proposed scheme			Khan, Masood & Alghafis (2020)			Munir et al. (2020)		
	**Red**	**Green**	**Blue**	**Red**	**Green**	**Blue**	**Red**	**Green**	**Blue**
**Baboon**	7.9992	7.9993	7.9993	7.9992	7.9993	7.9990	7.9992	7.9993	7.9992
**Fruits**	7.9993	7.9993	7.9993	7.9990	7.9990	7.9989	7.9993	7.9992	7.9994
**House**	7.9993	7.9994	7.9993	7.9991	7.9993	7.9991	7.9971	7.9974	7.9971
**Airplane**	7.9993	7.9993	7.9992	7.9993	7.9991	7.9992	7.9993	7.9993	7.9992
**Lena**	7.9993	7.9994	7.9993	7.9968	7.9986	7.9984	7.9992	7.9993	7.9993
**Peppers**	7.9994	7.9993	7.9994	7.9995	7.9993	7.9992	7.9992	7.9993	7.9993

The difference and assumption of the NPCR for two preferably encipher images are contrasted and the existing all-around plans in writing. The proposed algorithm NPCR results show that most of the values are close to 100 percent, as shown in Table 11. Similarly, the UACI test is compared between two optimally enciphered images. If the image encryption technique has a UACI score that is either too low or too high, it fails the test. Table 16 shows the comparison of statistical analysis with literature.

**Table 16:** Analysis comparison of proposed scheme with literature.

**Analysis**		Zhang (2021)	Sani, Behnia & Akhshani (2021)	Proposed
**Entropy**		7.997	7.9972	7.9998
**UACI**		33.42	32.51	33.47
** NPCR**		99.504	99.86	99.623
**Correlation**	Horizontal	0.0009	0.0012	0.0001
	Diagonal	−0.0288	0.0033	0.0041
	Vertical	−0.0113	0.0019	0.0013

## Conclusions

With the advancement of information technology, image data has become the primary content of network data transfer. The advancement of image encryption technology brings image information theft technologies. We need stronger S-boxes for image encryption algorithms to keep up with advancing information theft technologies. A novel strong substitution box for image encryption technique, Mandelbrot set, and Chen’s chaotic substitution permutation network is presented in this article. Our study’s main contribution is a chaotic-cryptographic system that can stop differential, linear, and brute force invasions while simultaneously improving the Shannon entropy of cipher images. We used several analyses on our proposed cryptosystem to validate the proposed approach experimentally, including non-linearity, BIC, SAC, histogram analysis, entropy, NPCR, and UACI. In the future, we propose adapting the current approach to operate with additional types of data such as audio and video.

## Supplemental Information

10.7717/peerj-cs.892/supp-1 Supplemental Information 1 The source code used for S-box generation

10.7717/peerj-cs.892/supp-2 Supplemental Information 2 The source code for image encryption

## Additional Information and Declarations

### Data Availability

The following information was supplied regarding data availability: The data is available at https://sipi.usc.edu/database/. The images in the USC Image Database are intended for research purposes.
https://sipi.usc.edu/database/copyright.php.
